# Nectin-4-Directed Therapeutic Repositioning Augments Antitumor Efficacy in Epithelial Ovarian Carcinoma

**DOI:** 10.34133/cancomm.0057

**Published:** 2026-09-30

**Authors:** Katherine Xiaoyan Zhong, Runying Long, Jiangnan He, Yanqing Li, Ruiqian Zhang, Ling Shan Hung, Can Cui, Cheng Bao, Crystal Tang, Zhangping Xiao, Michelle Kwan Yee Siu, Tina Na Wei, Stephanie Si Liu, Yingjie Xu, Huijuan Yang, Kui Liu, Bryan Cho Wing Li, Haonan Lu, Karen Kar Loen Chan

**Affiliations:** ^1^ Department of Obstetrics and Gynaecology, Li Ka Shing Faculty of Medicine, The University of Hong Kong, Hong Kong SAR, P. R. China.; ^2^ Materials Innovation Institute for Life Sciences and Energy, The University of Hong Kong–Shenzhen Institute of Research and Innovation, Shenzhen, P. R. China.; ^3^ Department of Chemistry, Imperial College London, London, UK.; ^4^ Department of Biochemistry and Molecular Cell Biology, Shanghai Key Laboratory for Tumor Microenvironment and Inflammation, Shanghai Jiao Tong University School of Medicine, Shanghai, P. R. China.; ^5^ Department of Gynecologic Oncology, Fudan University Shanghai Cancer Center, Shanghai, P. R. China.; ^6^ Department of Oncology, Shanghai Medical College, Fudan University, Shanghai, P. R. China.; ^7^ Shenzhen Key Laboratory of Fertility Regulation, Center of Assisted Reproduction and Embryology, The University of Hong Kong–Shenzhen Hospital, Shenzhen, P. R. China.; ^8^ Department of Medicine, Li Ka Shing Faculty of Medicine, The University of Hong Kong, Hong Kong SAR, P. R. China.; ^9^ Centre of Cancer Medicine, Li Ka Shing Faculty of Medicine, The University of Hong Kong, Hong Kong SAR, P. R. China.; ^10^ Department of Obstetrics and Gynaecology, The University of Hong Kong–Shenzhen Hospital, Shenzhen, P. R. China.

Antibody–drug conjugates (ADCs) represent a transformative class of targeted therapeutics designed to deliver potent cytotoxic payloads directly to tumor cells via antigen-specific monoclonal antibodies. The recent approval of mirvetuximab soravtansine (MIRV) for folate receptor alpha (FRα)-positive, platinum-resistant epithelial ovarian cancer (EOC) validates this approach [1]. However, only 36% of EOC patients are FRα-high and thus eligible to benefit from MIRV, leaving a substantial population without an ADC-based option [2,3]. Nectin cell adhesion molecule 4 (Nectin-4) expression is elevated in several solid malignancies, and a Nectin-4-directed ADC, enfortumab vedotin (EV), is approved for advanced urothelial cancer [4]. Although preliminary studies have suggested Nectin-4 up-regulation in EOC, a rigorous preclinical evaluation of Nectin-4-directed ADC efficacy in EOC is lacking [5].

We first examined *NECTIN4* expression in EOC using our institutional RNA-sequencing cohort (*n* = 420). *NECTIN4* mRNA expression was elevated in all examined EOC subtypes compared to normal ovarian tissues, with a heterogeneous expression pattern across subtypes (Fig. 1A). This pattern was corroborated at both the transcript and protein levels. In The Cancer Genome Atlas-Ovarian Cancer cohort, *NECTIN4* mRNA was markedly higher in EOC than in nonmalignant ovarian tissue, although lower than in bladder urothelial carcinoma (Fig. S1A). At the protein level, Nectin-4 immunohistochemistry in 2 tissue microarrays (155 EOC and 15 normal) showed substantially stronger expression in EOC than in normal ovarian tissue (Fig. 1B and Fig. S1B and C; Table S1). These findings support further investigation of Nectin-4 as a therapeutic target in EOC. Immunofluorescence and flow cytometry of EOC cell lines established models with high (OVISE, OAW28, and COV413A) and low (FT190, OVSAHO, and OVCAR8) membrane Nectin-4 expression, mirroring patient heterogeneity and providing a representative in vitro system (Fig. 1C and Fig. S1D to F).

**Fig. 1.**
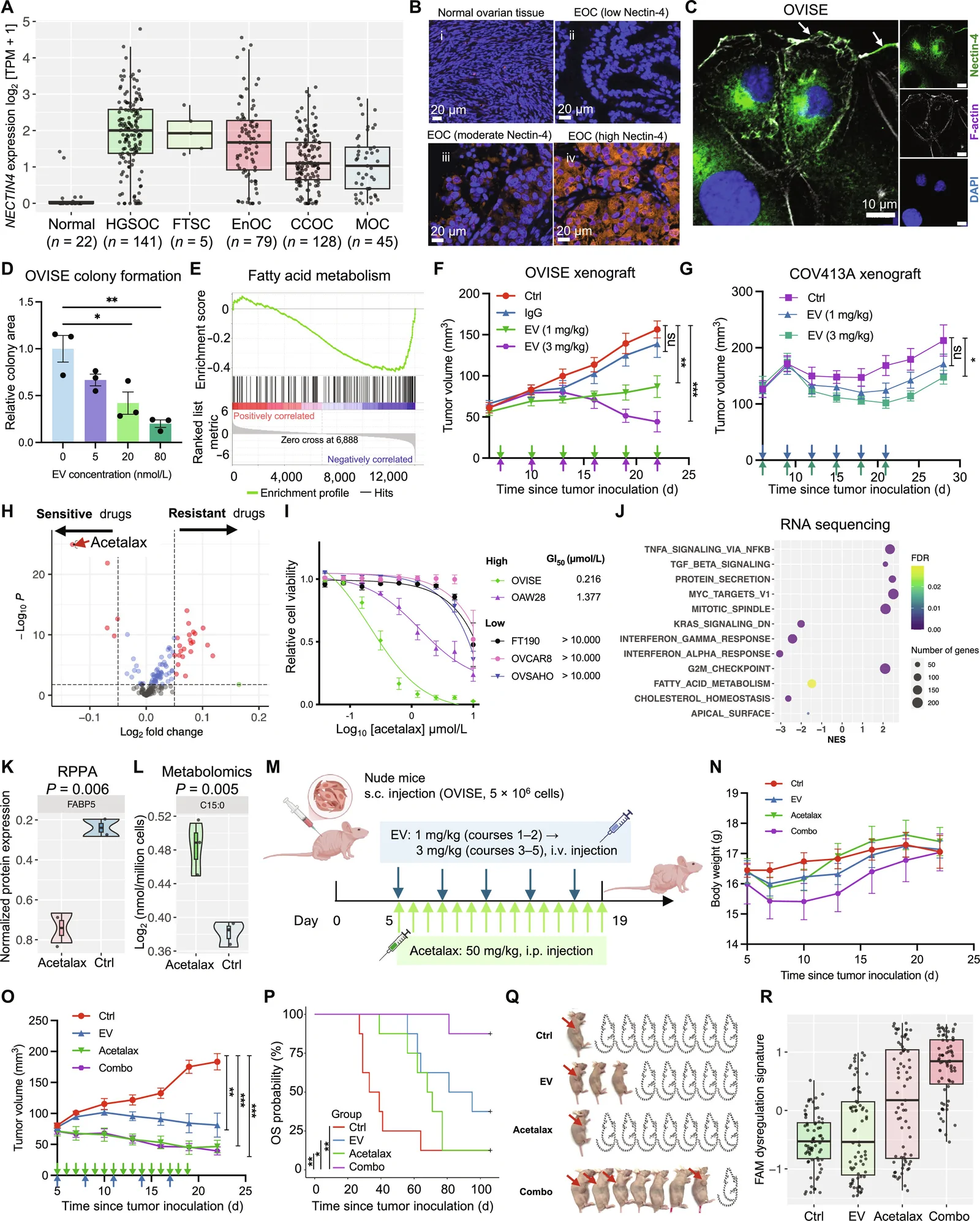
Nectin-4 as a therapeutic target in EOC. (A) Comparison of *NECTIN4* expression levels across normal ovarian tissues and EOC subtypes from the Queen Mary Hospital cohort. Data are derived from RNA-sequencing results of 420 patient samples. *X*-axis, normal ovarian tissues and EOC subtypes including HGSOC, FTSC, EnOC, CCOC, and MOC; *y*-axis, log_2_ ratio of transcript per million (TPM) + 1. Comparison between HGSOC and bladder urothelial tumors (BLCA) is shown in Fig. S1A and B. (B) Representative images of immunohistochemical staining of Nectin-4 in human normal ovarian tissues and EOC derived from tissue microarrays. (i) Normal ovarian tissue; (ii to iv) EOC with different expression levels of Nectin-4. Scale bar, 20 μm. Quantification of Nectin-4 expression is summarized in Table S1. (C) Representative confocal microscopy images illustrating the subcellular localization of Nectin-4 in the OVISE cell line. Cells were stained with anti-Nectin-4 antibody (green), F-actin of the cytoskeleton (gray), and DAPI (blue). Arrows highlight cells exhibiting membrane-specific positive staining of Nectin-4. Scale bar, 10 μm. Confocal microscope images of OAW28, as well as negative controls, are shown in Fig. S1E and F. (D) Quantification of colony formation in OVISE cell line in response to 0–80 nmol/L EV for 7 d. The colony area was quantified using ImageJ. Data are expressed as fold-change relative to control and presented as mean ± SEM (*n* = 3). Representative images are shown in Fig. S2B and C. (E) Depletion of fatty acid metabolism pathway in OVISE cells treated with 40 nmol/L EV for 3 d. A summary of all pathways is shown in Fig. S1F. (F and G) Tumor volume in OVISE-derived (F) and COV413A-derived (G) models treated with control or EV, measured every 3 d from baseline until day 28 post-inoculation. Ctrl, saline; IgG, isotype IgG control. EV was administered at 1 mg/kg or 3 mg/kg every 3 d. Statistical significance was determined by comparing each treatment group with the saline control. Detailed treatment schedules for the OVISE and COV413A models are provided in Fig. S3A and B. Arrows indicate the treatment schedule for EV at 1 mg/kg (top) or 3 mg/kg (bottom). Body-weight changes throughout the 2 models are shown in Fig. S3C and D. Changes in individual tumor volume are provided in Fig. S3E and F. COV413A-derived tumor weight at endpoint is shown in Fig. S3G. (H) Volcano plot illustrating the association between *NECTIN4* expression and sensitivity to 175 small-molecule drugs in 22 EOC cell lines from the DepMap database. The area under the curve of each drug was compared between *NECTIN4*-high and *NECTIN4*-low cell lines using a moderated *t* test. The horizontal dotted line indicates a false discovery rate <0.05. Association between acetalax sensitivity and Nectin-4 expression in EOC cell lines is also shown in Fig. S4A and B. (I) Dose–response curve of 5 EOC-relevant cell lines treated with acetalax for 7 d. Data are presented as mean ± SEM from 3 independent experiments. Based on their relative Nectin-4 expression levels (Fig. S1D), cell lines are classified as Nectin-4 high or low, with corresponding growth inhibition 50% (GI_50_) values shown alongside each cell line. (J) Summary of 12 differential pathway enrichments in OVISE cells treated with 100 nmol/L of acetalax for 3 d. Data are presented as the normalized enrichment score (NES), and statistical significance (false discovery rate) of enrichment is indicated in colors. A full list of the dysregulated pathways is shown in Table S5. Top altered genes in fatty acid metabolism and apoptosis pathways are shown in Fig. S5A. (K) Violin plot showing altered protein expression of FABP5, a protein involved in fatty acid metabolism pathways, as measured by RPPA, in OVISE cells treated with 100 nmol/L acetalax for 3 d. *Y*-axis, normalized protein expression. (L) Violin plot from targeted metabolomics profiling illustrating dysregulation of C15:0 in OVISE cells treated with 100 nmol/L acetalax for 3 d. *Y*-axis, nmol of the fatty acid per million cells in log_2_ ratio. Other dysregulated fatty acids in acetalax-treated OVISE are shown in Fig. S5C and D. (M) Work scheme of the in vivo combination treatment in the OVISE model. Tumors stabilized on day 5 after inoculation and were chosen as the baseline. A total of 5 courses of EV were given intravenously: dosage started with 1 mg/kg every 3 d for the first 2 courses and was elevated to 3 mg/kg for 3 courses. A total of 15 courses of acetalax at 50 mg/kg were given daily. Single-agent groups received EV or acetalax alone, while the combination group received both agents on the same schedules. Saline was used in the control groups. (N) Body weight of mice bearing OVISE-derived tumors, monitored from treatment baseline (day 5) to day 22 after tumor inoculation. (O) OVISE-derived tumor volume was measured every 3 d from baseline until day 22 post-inoculation. EV (1 mg/kg or 3 mg/kg, every 3 d) and acetalax (50 mg/kg, daily) were administered as single agents or in combination according to the schedule outlined in Fig. 1M. Statistical significance was determined by comparing each treatment group with the saline control. Arrows indicate the treatment schedule for acetalax (top) and EV (bottom). Changes in individual tumor volume are shown in Fig. S7E. The tumor volume changes during the complete 79-d period are shown in Fig. S7F. (P) Kaplan–Meier survival curves comparing the overall survival of mice bearing OVISE-derived tumors from the control group and the treatment groups (*n* = 8 for each group at baseline). *P* values were determined by the log-rank test comparing the combo group with the control group, the EV monotherapy group, or the acetalax monotherapy group, adjusted for 3 comparisons. Treatment schedule outlined in Fig. 1M. (Q) Diagram showing all surviving mice bearing OVISE-derived tumors at the study endpoint on day 99 post-inoculation. All mice euthanized before the study endpoint for tumor ulceration or tumor oversize are illustrated in a dashed line. Mice bearing tumors are depicted with a red arrow. (R) Fatty acid metabolism dysregulation signature enriched in residual tumors from the OVISE-derived xenograft model treated with saline, EV, acetalax, or combination. Data are expressed as fold-change relative to control and presented as mean ± SEM from 3 independent experiments. Statistical significance is determined by an unpaired two-tailed Student *t* test (ns, not significant; *P* > 0.05, *P < 0.05, **P < 0.01, ***P < 0.001). NECTIN4, nectin cell adhesion molecule 4; EOC, epithelial ovarian cancer; HGSOC, high-grade serous ovarian carcinoma; FTSC, fallopian tube serous carcinoma; EnOC, endometrioid ovarian carcinoma; CCOC, clear cell ovarian carcinoma; MOC, mucinous ovarian carcinoma; DAPI, 4',6-diamidino-2-phenylindole; EV, enfortumab vedotin; SEM, standard error of the mean; FABP5, fatty acid binding protein 5; C15:0, pentadecanoic acid; OS, overall survival; s.c., subcutaneous; i.v., intravenous; i.p., intraperitoneal.

We next evaluated the cytotoxic efficacy of EV using in vitro and in vivo EOC models. Cell lines with high Nectin-4 expression (OVISE, OAW28, and COV413A) exhibited nanomolar-range sensitivity (growth inhibition 50% [GI_50_]: 3.033 to 7.856 nmol/L), whereas low-expressing cell lines (FT190, OVCAR8, and OVSAHO) exhibited reduced sensitivity (GI_50_ > 70.000 nmol/L) (Fig. S2A). Functional assays confirmed that EV dose-dependently inhibited clonogenic survival and induced apoptosis in sensitive OVISE, OAW28, and COV413A cells (Fig. 1D and Fig. S2B to E). Gene set enrichment analysis of EV-treated OVISE cells revealed activation of p53 signaling and pronounced down-regulation of the fatty acid metabolism (FAM) pathway, suggesting rapid disruption of this critical cancer-supporting pathway (Fig. 1E and Fig. S2F; Table S2) [6,7]. The antitumor efficacy of EV was subsequently validated in vivo using 2 subcutaneous models derived from Nectin-4-high cell lines (OVISE and COV413A; Fig. 1F and Fig. S3A and B). Treatment with EV (3 mg/kg) markedly suppressed tumor growth in both models compared with the control arms and was well tolerated (Fig. S3C and D). Notably, EV monotherapy at 1 mg/kg in OVISE and 1 or 3 mg/kg in COV413A led to evident recurrence (Fig. 1F and Fig. S3E to G; Table S3), mirroring the clinical challenge of ADC resistance and directly motivating us to identify combination partners that enhance the efficacy of EV [8].

To address this challenge, we aimed to identify additional agents that can selectively eradicate Nectin-4-high EOC. An in silico analysis of the Genomics of Drug Sensitivity in Cancer drug sensitivity database across 22 EOC cell lines revealed a positive correlation between Nectin-4 expression and sensitivity to oxyphenisatin acetate (*P* < 0.001) (Fig. 1H and Fig. S4A; Table S4). Oxyphenisatin acetate, also known as acetalax, was originally developed as an oral stimulant laxative [9]. Consistent with the in silico findings, Nectin-4-high cell lines (OVISE and OAW28) exhibited sensitivity to acetalax in the sub-micromolar range (GI_50_: 0.216 to 1.377 μmol/L), whereas Nectin-4-low cell lines (FT190, OVCAR8, and OVSAHO; GI_50_ > 10.000 μmol/L) showed reduced sensitivity to acetalax (*P* = 0.030) (Fig. 1I and Fig. S4B). Functionally, 500 nmol/L acetalax inhibited colony formation and induced apoptosis in cells with high Nectin-4 expression, with no effect on Nectin-4-low cell lines (Fig. S4C to F).

We next investigated the potential mechanism underlying acetalax sensitivity in Nectin-4-high cells. RNA-seq and reverse phase protein array analysis of acetalax-treated OVISE cells confirmed induction of apoptosis gene set and related proteins—including diablo IAP-binding mitochondrial protein (DIABLO/Smac) (Table S5; Fig. S5A and B)—and down-regulation of FAM pathway gene set and key metabolic proteins such as fatty acid binding protein 5 (Table S5; Fig. 1J and Fig. S5A). Targeted metabolomics profiling further demonstrated dysregulation of cellular fatty acids, including C15:0, C16:0, and C17:0, upon acetalax treatment (Fig. 1L and Fig. S5C and D). *NECTIN4* knockdown did not alter acetalax sensitivity, but *NECTIN4* predicted acetalax response across 520 cell lines and correlated with TRPM4, the mechanistic target of acetalax [9], at both transcript and protein levels, indicating that Nectin-4 is a potential predictive biomarker rather than a mechanistic mediator (Fig. S6).

The shared biomarker and phenotype provided a strong rationale for testing combination therapy. In vitro, the EV–acetalax combination demonstrated synergistic cytotoxicity in both Nectin-4-high EOC and urothelial cancer cells (Fig. S7A to D). We evaluated this combination in vivo using the OVISE xenograft model treated with saline, EV (1 to 3 mg/kg, escalated), acetalax (50 mg/kg), or the combination (Fig.1M). The regimen was well tolerated, with only moderate changes in body weight observed across all groups (Fig. 1N). During the active treatment phase, the combination did not further reduce tumor volume beyond what was achieved by each monotherapy alone, likely due to the high initial efficacy of both agents (Fig. 1O and Fig. S7E). However, combination treatment markedly prolonged overall survival relative to control (hazard ratio [HR] = 0.057; 95% confidence interval [CI], 0.003 to 0.347; *P* = 0.003), EV alone (HR = 0.142; 95% CI, 0.007 to 0.889; *P* = 0.037), and acetalax alone (HR = 0.057; 95% CI, 0.003 to 0.347; *P* = 0.003; log-rank, Holm-adjusted for three comparisons) (Fig. 1P and Fig. S7F; Table S6). Additionally, we observed a similar enhancing effect with bisacodyl, a structurally related laxative that remains in clinical use. The combination of 100 mg/kg bisacodyl and 1.25 mg/kg EV substantially reduced tumor volume compared with the control or single-agent treatments (Fig. S8A to C). This pattern is consistent with the notion that co-targeting Nectin-4-high EOC with acetalax or its derivative may enhance the durability of EV response. Transcriptomic analysis of residual tumors at endpoint showed the most profound dysregulation of FAM pathways in the combination group, suggesting an amplified effect on this vulnerability (Fig. 1R).

The link between Nectin-4 expression and acetalax sensitivity, though not mechanistically causal, presents a valuable biomarker strategy. As both agents have established human safety profiles [4,5,9,10], this combination offers a potential pathway for rapid clinical translation. Importantly, despite hepatotoxicity with chronic use (>6 months), short-term acetalax caused only mild liver inflammation in mice (Fig. S8D), suggesting clinical feasibility with monitoring. We observed a differential in vivo response to EV between OVISE and COV413A, which likely reflects multiple factors: higher membrane Nectin-4 density in OVISE that may promote ADC internalization; slower proliferation of COV413A that may reduce sensitivity to the MMAE payload; and the larger starting volume of COV413A tumors (100 vs. 50 mm^3^). This heterogeneity introduces uncertainty in predicting patient benefit and underscores the need for prospective validation. Additional limitations include the need for patient-derived organoid studies to confirm Nectin-4 as a predictive biomarker for acetalax sensitivity and the requirement for functional rescue experiments to establish causality in fatty acid metabolism disruption and acetalax response. Finally, although the EV doses used are lower than the doses reported in other xenograft models [5], we acknowledge that a future clinical trial is required to evaluate equivalent weight-based dosing in human EOC patients. In conclusion, this study provides preclinical evidence supporting Nectin-4 as a therapeutic target in EOC and suggests that combination with acetalax may represent a promising strategy to enhance ADC efficacy, warranting further investigation.

## Ethics Approval and Consent to Participate

The retrospective analysis of human participant data was approved by the Institutional Review Board of the University of Hong Kong/Hospital Authority Hong Kong West Cluster (HKU/HA HKW IRB: UW 11-298). Written informed consent was obtained from all participants. All animal experiments were approved by the Committee on the Use of Live Animals in Teaching and Research of the University of Hong Kong (CULATR: 24-132) or Signet Therapeutics Institutional Animal Care and Use Committee (SIGX-2025-140) and were conducted in accordance with institutional guidelines.

## Data Availability

All data supporting the findings of this study are available in the main text and the supplementary files. RNA-sequencing, reverse phase protein array, and mass spectrometry data are available through Mendeley Data (doi:10.17632/f78h88y52d).
