# Supplementary material for: Nectin-4-Directed Therapeutic Repositioning Augments Antitumor Efficacy in Epithelial Ovarian Carcinoma
Source: Cancer Commun (Lond). 2026 Sep 30;46:0057. doi: 10.34133/cancomm.0057 (PMC13624863; doi:10.34133/cancomm.0057)
Supplement: Supplementary file 1 [file cancomm.0057.f1.zip › CANCOMM-D-26-00072_SuppleM_clean.pdf]

Supplementary Materials for

Nectin-4-directed Therapeutic Repositioning Augments Anti-tumor Efficacy in Epithelial Ovarian Carcinoma

Katherine Xiaoyan Zhong[1, 2], Runying Long[1, 2], Jiangnan He[1, 2], Yanqing Li[1, 2], Ruiqian Zhang[1], Ling Shan Hung[1], Can Cui[1, 2], Cheng Bao[1, 2], Crystal Tang[1], Zhangping Xiao[3], Michelle Kwan Yee Siu[1], Tina Na Wei[1], Stephanie Si Liu[1], Yingjie Xu[4], Huijuan Yang[5, 6], Kui Liu[1, 7], Bryan Cho Wing Li[1, 8, 9 *], Haonan Lu[1, 2, 10, *], Karen Kar Loen Chan[1, 10, *]

[1] Department of Obstetrics and Gynaecology, Li Ka Shing Faculty of Medicine, The University of Hong Kong, Hong Kong SAR, P. R. China

[2] Materials Innovation Institute for Life Sciences and Energy, The University of Hong Kong-Shenzhen Institute of Research and Innovation, Shenzhen, P. R. China

[3] Department of Chemistry, Imperial College London, London, United Kingdom

[4] Department of Biochemistry and Molecular Cell Biology, Shanghai Key Laboratory for Tumor Microenvironment and Inflammation, Shanghai Jiao Tong University School of Medicine, Shanghai, P. R. China

[5] Department of Gynecologic Oncology, Fudan University Shanghai Cancer Center, Shanghai, P. R. China

[6] Department of Oncology, Shanghai Medical College, Fudan University, Shanghai, P. R. China

[7] Shenzhen Key Laboratory of Fertility Regulation, Center of Assisted Reproduction and Embryology, The University of Hong Kong-Shenzhen Hospital, Shenzhen, P. R. China

[8] Department of Medicine, Li Ka Shing Faculty of Medicine, The University of Hong Kong, Hong Kong SAR, P. R. China

[9] Centre of Cancer Medicine, Li Ka Shing Faculty of Medicine, The University of Hong Kong, Hong Kong SAR, P. R. China

[10] Department of Obstetrics and Gynaecology, The University of Hong Kong-Shenzhen Hospital, Shenzhen, P. R. China

*Address correspondence to: K.K.L.C., kklchan@hku.hk; H.L., haonanlu@hku.hk; B.C.W.L., bryanli@hku.hk.

Supplementary Materials and Methods

Cell lines and reagents

Human ovarian cancer cell lines OVISE and OVSAHO were obtained from the Japanese Collection of Research Bioresources (JCRB); OVCAR8 and OAW28 were obtained from AcceGen Biotech, US; COV413A was obtained from European Collection of Authenticated Cell Cultures (ECACC). Human fallopian tube epithelial cell line FT190 [1] was a kind gift from Prof. Ronny Drapkin at Dana-Farber Cancer Institute; Human urothelial carcinoma cell lines SW780, RT-112, and HT-1376 were obtained from Immocell Biotechnology Co., Ltd.; RT-4, UMUC3, HUC-1, T24, EJ1, and J82 were kind gifts from Prof. Rong Na at the University of Hong Kong. Cell line authentication was conducted within one year of study completion.

All cell lines were cultured in RPMI 1640 Medium, DMEM, McCoy's, MEM, and L15 with 4 mmol/L L-glutamine (Life Technologies) and supplemented with 10% FBS. Cells were maintained under conditions of 37°C with 5% carbon dioxide and passaged with 0.05% Trypsin-EDTA once reaching 70% confluency. Mycoplasma was monitored every 2 months by the Centre for PanorOmic Sciences (CPOS) at the Li Ka Shing Faculty of Medicine, the University of Hong Kong.

Enfortumab vedotin-ejfv (PADCEV; EV) was purchased from Astellas Pharma Inc. Acetalax (oxyphenisatin acetate, HY-101714) was purchased from MedChemExpress. Bisacodyl (Dulcolax Fenilaxan, T6414) was purchased from TargetMol.

Immunohistochemical staining

Two tissue microarrays (TMA) containing single core of 1.5mm from 155 primary ovarian cancer cases (low-grade serous ovarian carcinoma (LGSOC): *n* = 20; high-grade serous ovarian carcinoma (HGSOC): *n* = 77; endometrioid ovarian carcinoma (EnOC): *n* = 19; clear cell ovarian carcinoma (CCOC): *n* = 20; mucinous ovarian carcinoma (MOC): *n* = 19) and 15 normal adjacent ovarian tissue was obtained from the Tissue Array (OV1006 and OV8011a). TMA were prepared with Opal Polaris 7 Color IHC Detection Kit (AKOYA, NEL861001KT). TMA was then incubated with 3% hydrogen peroxide to block endogenous peroxidase activity and blocked with 3% BSA for 10 minutes at room temperature. Slide was stained with anti-Nectin-4 antibody (R&D Systems, AF2659) overnight at 4°C. After washing, the slide was stained with boost immunohistochemistry (IHC) detection reagent (Cell Signaling, 63707) for 30 minutes at room temperature. The slide was then washed and stained with an opal fluorophore. The previous steps were repeated for the next target with anti-folate receptor alpha antibodies (Leica Biosystems, NCL-L-FRα). Slide was then dehydrated, and cover slipped with antifade mountant (Invitrogen, P36970).

Flow cytometry

Culture cells were detached by 0.05% Trypsin-EDTA, washed with PSS (PBS containing 2% FBS), and incubated with anti-Nectin-4 antibody (R&D Systems, AF2659) for 30 minutes at 4°C. Cells were washed and incubated with donkey anti-goat IgG (Invitrogen, A-21447) for 30 minutes at 4°C. After washing, cells were fixed with 4% PFA and run on Agilent NovoCyte Advanteon BVR at CPOS.

Immunofluorescence microscopy

Culture cells were seeded on chambered coverslips (ibidi μ-Slide) in complete culture medium and allowed to attach for 24 hours. The cells were washed with PSS and fixed in 4% PFA, followed by blocking with PSS. The cells were then stained with the primary antibody and the secondary antibodies mentioned in the flow cytometry. After washing, coverslips were mounted with mounting medium (Sigma, F6057). The cells were then imaged by a confocal microscope (Carl Zeiss LSM800).

Cytotoxicity assay and drug synergistic effect

Five hundred to 2,500 cells per well were seeded in 96-well plates and allowed to attach for 24 hours. Cells were then treated with serially diluted EV, acetalax, or bisacodyl for 7 days. After treatment, cellular viability was measured by the MTT assay (Invitrogen, M6494) following the manufacturer's protocol. Cell viability was determined by measuring optical density at 570 nm (Tecan). The Growth Inhibition 50% (GI_{50}) was calculated in GraphPad Prism. The synergistic effect of the drug combination was analyzed with the Combenefit Software [2] using the HSA model.

Apoptosis assay

Cell apoptosis was determined by the Annexin V assay kit (Invitrogen, V13241). A total of 1.5×10^5 cells were seeded in 6-well plates and incubated for 24 hours to allow attachment. Cell apoptosis was induced by treating with 0–80 nmol/L EV or 0–500 nmol/L acetalax. Cells were then harvested after 3 days of incubation and washed with PBS. Cells were resuspended and incubated for 15 minutes at room temperature with annexin-binding buffer containing Annexin V Alexa Fluor 488 and Propidium Iodide. After incubation and adding binding buffer, stained cells were then analyzed by flow cytometry. The percentage of apoptotic cells was quantified by the FlowJo FlowJ™ v10.8 Software (BD Life Sciences).

Clonogenic assay

Approximately 3×10^4 to 4×10^4 cells were seeded in 12-well plates and incubated for 24 hours to allow attachment. Cells were treated with 0–80 nmol/L EV or acetalax for 7 days. After washing with PBS, cells were stained with 0.5% crystal violet solution (Sigma) for 30 minutes. Stained cells were then washed and dried at room temperature. Cell colonies were counted by area using ImageJ2 v2.14 Software.

***siRNA* knockdown**

Approximately 2×10^5 cells were seeded in 6-well plates and allowed to attach for 24 hours. The gene knockdown was performed by transfecting siRNA of *NECTIN4* (IDT, 110456905 (CCAAACUCUUCUUAGUCUGAAAUCT); 110456908 (CAAGGGAUUCUCAGGUCACUGUGGA); 110456911 (GUGGUGCUCAUGUCCCGAUACCATC)). *siRNA* delivery was induced using SilentFect lipid reagent (Bio-Rad, 1703361) following the manufacturer's protocol. After 6 hours of incubation with *siRNA*, the medium was refreshed with full culture medium. After 24 hours of incubation, the transfected cells were then ready for flow cytometry, cytotoxicity assay, and Western blotting.

***In vivo* study**

All experimental protocols were approved by the Centre for Comparative Medicine Research (CCMR) of the University of Hong Kong (HKU, approval number: 24-132) or Signet Therapeutics Institutional Animal Care and Use Committee (SIGX-2025-140).

The therapeutic efficacy of EV as monotherapy and in combination was evaluated in female Balb/c nude mice (5–6 weeks old; supplied by CCMR HKU). Mice were inoculated subcutaneously with a suspension of 5×10^6 to 1×10^7 cells in 100% Matrigel basement membrane matrix (Corning, 354234). OVISE and COV413A successfully formed stable and progressive tumors in nude mice. OAW28 line failed to establish xenograft models in both nude mice and NSG mice, as measurable tumors regressed spontaneously after inoculation within 3–5 weeks. The baseline measurement was established when tumor volumes stabilized within the range 50-150 mm^3. Due to differences in intrinsic growth kinetics between the two models, treatment was initiated at 50 mm^3 for OVISE and 100 mm^3 for COV413A to ensure tumors were established and palpable in both models. Mice were randomized into treatment groups of 8 per group.

EV or human IgG control (BioXCell, BE0297) was diluted in sterile saline and administered via intravenous tail vein injection at doses of 1 or 3 mg/kg once every three days. Acetalax and bisacodyl were diluted in a vehicle of 10% DMSO, 40% PEG300, 5% Tween-80, and 45% sterile saline and administered via intraperitoneal injection at 50–100 mg/kg once daily. In the study of the anti-tumor efficacy of monotherapy, 6 courses of EV were given to the OVISE-bearing mice, and 5 courses of EV were given to the COV413A-bearing mice. For the combined treatment experiment, a total of 5 courses of EV and 15 courses of acetalax were given to the mice. In extended experiments examining the combined treatment of EV and bisacodyl, 2 courses of EV and 10 courses of bisacodyl were given to the mice.

Tumor volume and body weight of mice were measured every three days throughout the experiment. The tumor was measured by digital calipers, and the total volume was calculated with the formula $V = (\pi / 6) \times \text{Length} \times \text{Width} \times \text{Height}$. Mice were euthanized and considered reaching the study endpoint when tumor ulceration was observed, tumor length exceeded 12 mm, or they exhibited signs of significant distress or morbidity. The overall survival data were collected as a secondary endpoint in the study of combined treatment. Tumor recurrence was defined as a ≥30% increase

in tumor volume from the nadir (the smallest volume achieved post-treatment), confirmed on two consecutive measurements taken 6 days apart, following initial tumor regression or stabilization. Additionally, the overall survival endpoint was defined as the time when any humane endpoint was reached, including tumor oversize or tumor ulceration.

RNA sequencing

Approximately 1 × 10^5 OVISE cells were seeded on a 6-well plate in complete culture medium and allowed to attach for 24 hours. The cells were treated with EV (40 nmol/L) or acetalax (100 nmol/L) for 3 days. Cells were harvested after incubation, and RNA was extracted from the cell pellet by the RNA extraction kit (Qiagen; 74136). Purified RNA samples or fresh frozen residual tumors were sent to BGI Genomics for library preparation and subsequent RNA sequencing as previously described. Briefly, a eukaryotic strand-specific mRNA library was prepared from total RNA samples enriched for polyadenylated mRNA using oligo dT beads. The mRNA was fragmented into small pieces, and first-strand cDNA synthesis was performed using random primers, followed by second-strand synthesis with dUTP instead of dTTP to maintain strand specificity. The cDNA was then end-repaired, 3' adenylated, and adaptors ligated before PCR amplification. The resulting library underwent quality control, circularization, and amplification to produce DNA nanoballs (DNBs), which were sequenced on the DNBSEQ platform with PE100 reads. Raw sequencing data were filtered for adapter contamination, low-quality reads, short reads (<100 bp), reads containing high N content (>1%), and reads with polyX sequences (>50 bp) using SOAPnuke (https://github.com/BGI-flexlab/SOAPnuke) with specified parameters, ensuring high-quality clean reads for downstream analysis.

Raw FASTQ data were aligned to the human GRCh38 reference genome using STAR 2.7.3a (https://github.com/alexdobin/STAR) with default parameters, and gene counts were subsequently generated. Differential expression analysis was performed using DESeq2 (https://github.com/thelovelab/DESeq2). Pathway enrichment analysis with HALLMARK database was conducted on the differentially expressed genes using Gene Set Enrichment Analysis (GSEA; https://www.gsea-msigdb.org/gsea/index.jsp), applying default settings, including gene set permutation to compute normalized enrichment scores (NES) and FDR q-values. Pathways with an FDR < 0.05 were considered statistically significant.

Transcriptomic analysis of the TCGA-BLCA and TCGA-OV cohorts were performed using the GEPIA platform (http://gepia.cancer-pku.cn/index.html). Non-malignant samples from TCGA and GTEx were combined to form the normal control group; both datasets are uniformly reprocessed by the GEPIA pipeline (UCSC Xena Toil recompute), allowing them to be analyzed together. Expression values are given as $\log_2$(TPM + 1). Differential expression between tumor and normal groups was assessed by one-way ANOVA.

Reverse Phase Protein Array (RPPA)

Around 1 × 10^5 cells were seeded on a 6-well plate in complete culture medium and allowed to attach for 24 hours. The cells were treated with acetalax (100 nmol/L) for 3 days, and then harvested. Sample preparation for the assay was done according to the protocol provided by the RPPA core (MD Anderson Cancer Center, the University of Texas, US). Protein quantification was performed as previously described. Briefly, protein samples were serially diluted and spotted onto nitrocellulose slides arranged in a 4 × 12-pin array. Proteins were detected using specific antibodies, amplified with tyramide, and visualized with DAB staining. Images were captured with a Huron TissueScope scanner, and spot intensities were quantified with Array-Pro Analyzer. Relative protein levels were calculated by fitting dilution curves using the RPPA SPACE method [3] to determine half maximal effective concentration (EC50) values.

Metabolomics

Approximately 1.5 × 10^6 cells were seeded on a 150mm cell culture dish in complete culture medium and allowed to attach for 24 hours. The cells were treated with acetalax (100 nmol/L) for 3 days. Cells were harvested after incubation and were then submitted to the service provider for lipid profiling at CPOS. The mass spectrometry analysis was performed as previously described. [4] Briefly, samples were homogenized and metabolites extracted using a liquid-liquid extraction method. Specifically, 100 μL of chloroform containing 20 μg of C19:0 fatty acid internal standard (Sigma-Aldrich) was added to each sample, followed by five rounds of extraction with a 2:1 chloroform/methanol

mixture and sonication. After centrifugation, the supernatant was further purified through extraction with 0.73% NaCl and methanol. The resulting extract was then dried under a nitrogen stream at 45°C to prepare for transesterification.

For the analysis of fatty acid methyl esters (FAMEs), the dried extracts were subjected to transesterification by adding methanol and concentrated HCl, then heating at 100°C for 1.5 hours. The FAMEs were subsequently extracted with hexane and water, and a small aliquot was injected into an Agilent 7890B GC coupled with a 7010 Triple Quadrupole MS. Separation was performed on an Agilent DB-23 column with a defined temperature program, and data were acquired in both SCAN and SIM modes. Quantitative analysis involved calibration curves generated using internal standards, with analyte identification confirmed by characteristic fragment ion ratios.

Differential expression analysis was conducted using the 'limma' package (https://bioconductor.org/packages/release/bioc/html/limma.html) in R, which employs a moderated t-test to compare transcript levels between control and treated samples.

Western blotting

Cells were lysed using 1× lysis buffer (Cell Signaling Technology, 9803S) supplemented with 1× Protease Inhibitor Cocktail (Sigma-Aldrich, P2714) and 1 mmol/L phenylmethylsulfonyl fluoride (PMSF; Sigma-Aldrich, P7626) following the manufacturer's instructions. The cell lysate was centrifuged at 16,000 ×*g* for 20 min at 4°C, and the supernatant was collected. Protein samples were denatured by heating at 95°C for 10 min in reducing SDS sample buffer (Cell Signaling Technology, 7722) and subsequently separated by SDS-PAGE, followed by transfer onto a PVDF membrane. Membranes were blocked and incubated overnight at 4°C with the indicated primary antibodies: anti-TRPM4 rabbit monoclonal antibody (1:1,000; ABclonal, A25511) and anti-β-actin monoclonal antibody (BA3R) (1:5,000; Thermo Fisher Scientific, MA5-15739). Following primary antibody incubation, membranes were incubated with the appropriate HRP-linked secondary antibodies: Amersham ECL anti-rabbit IgG (1:5,000; Cytiva, NA934-1ML) or Amersham ECL anti-mouse IgG (1:5,000; Cytiva, NA931-1ML). Protein bands were visualized using an enhanced chemiluminescence (ECL) detection system. Each Western blotting image presented is representative of three independent experiments.

Prediction of acetalax sensitivity from NECTIN4 expression

NECTIN4 expression (log_2(TPM+1), short-read RNA sequencing) and acetalax dose–response data (area under the curve, GDSC2) were obtained from the DepMap portal (release 26Q1; https://depmap.org/portal/). Of 1,965 catalogued cell line models, 542 had complete data for *NECTIN4* expression, acetalax dose-response AUC and OncotreeLineage annotation; lineages represented by fewer than 10 cell lines were excluded, leaving 520 lines across 19 lineages. Cell lines were classified as acetalax-sensitive if the drug-response AUC was below 0.7 (39/520, 7.5%). Cell lines were assigned at random in equal proportion to a training or validation set (set.seed(12345); n = 270 each set), and a logistic regression model was fitted in the training set with *NECTIN4* expression as the sole predictor. Discrimination was assessed by the area under the receiver operating characteristic curve, with 95% confidence intervals estimated by the DeLong method. P-values were calculated using Mann-Whitney Test. All analyses were performed in R 4.3.3 using the pROC package (v1.18.5) and the verification package (v1.45).

Statistical analysis

Statistical analysis was performed using GraphPad Prism version 10.0 software. (GraphPad Software, Inc.). The two-tailed unpaired t-test was used to evaluate the difference between two groups, including colony formation inhibition and apoptotic rates in EOC cell lines treated with EV and acetalax. For time-to-event data, survival rates were analyzed using the Kaplan-Meier method, and differences between groups were assessed with the log-rank test. Statistical significance was defined as follows: * $P < 0.05$, ** $P < 0.01$, *** $P < 0.001$.

Supplementary Tables

Table S1. Immunohistochemistry scoring of Nectin-4 expression across epithelial ovarian cancer subtypes using two commercial tissue microarrays.

Table S2. Gene Set Enrichment analysis of EV-treated OVISE.

Table S3. Tumor volume of OVISE-derived xenograft model treated with EV.

Table S4. *NECTIN4* expression and matched acetalax sensitivity data in 540 human cancer cells from the DepMap database.

Table S5. Gene Set Enrichment analysis of acetalax-treated OVISE.

Table S6. Tumor volume of OVISE-derived xenograft model treated with EV, acetalax and combination.

Supplementary Figures

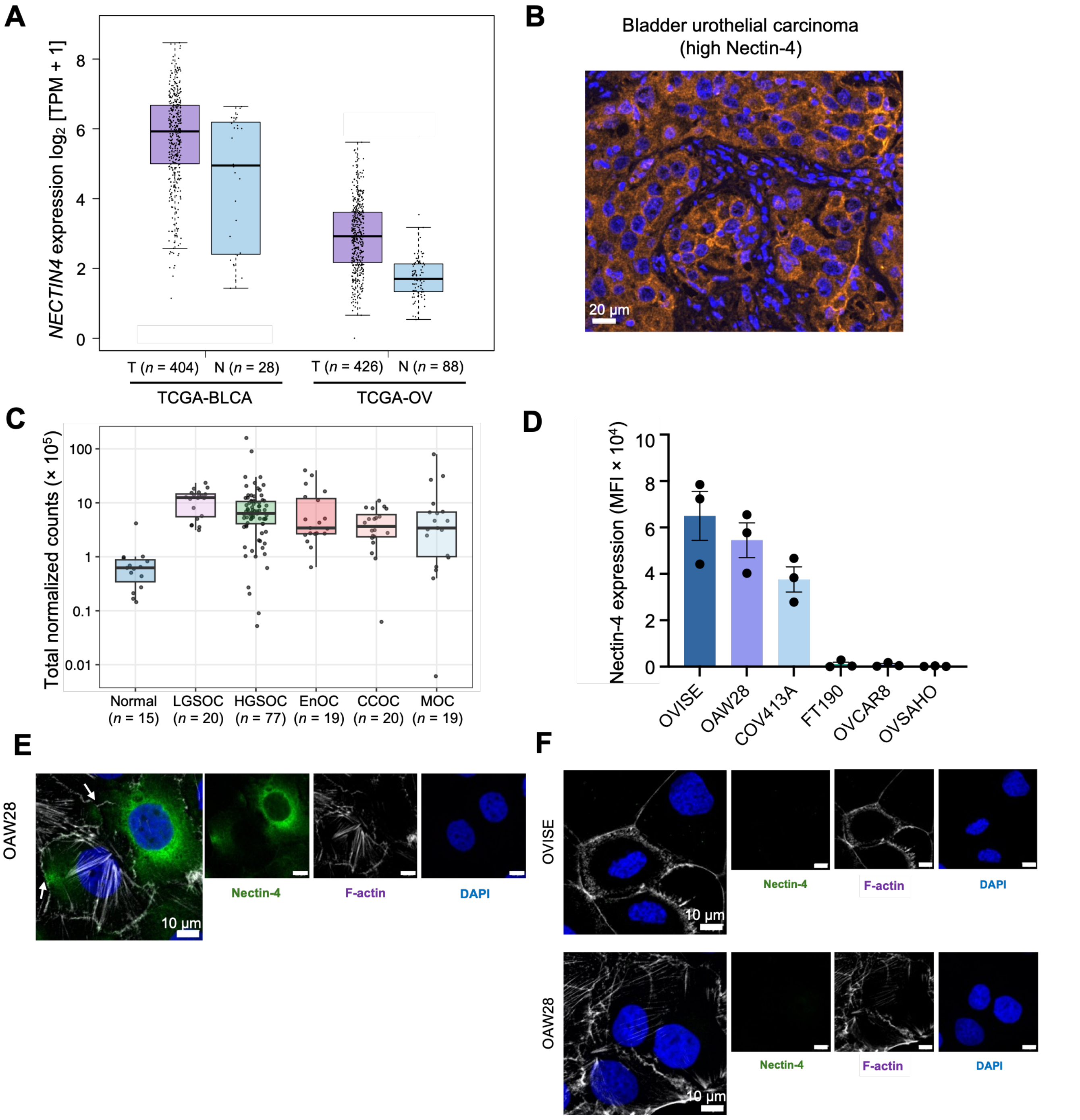

Fig. S1. Expression profile of Nectin-4 in epithelial ovarian cancer. (A) Comparison of *NECTIN4* mRNA expression levels between urothelial cancer and ovarian cancer tissue and their matched normal tissues (blue) from the TCGA cohort (TCGA-BLCA and TCGA-OV). X-axis, TCGA-BLCA, TCGA bladder urothelial carcinoma cohort; TCGA-OV, TCGA ovarian serous cystadenocarcinoma cohort; Y-axis, $\log_2$ ratio of transcript per million (TPM) + 1. **(B)** Representative images of immunohistochemical staining of high Nectin-4 expression in urothelial carcinoma tissue collected from Queen Mary Hospital. Scale bar, 20 µm. **(C)** Quantification of Nectin-4 protein expression in 80 primary ovarian cancer cases and 10 normal ovarian tissues assessed using tissue microarrays. Data presented as total normalized counts of tumors in each EOC subtype and normal ovarian tissues. **(D)** Flow cytometry analysis on the membrane protein expression of Nectin-4 in a panel of EOC-relevant cell lines, including OVISE, OAW28, COV413A, FT190, OVCAR8, and OVSAHO. Nectin-4 abundance in cells is expressed as the Mean Fluorescence Intensity (MFI) and presented as mean ± SEM of 3 biological replicates. **(E-F)** Representative confocal microscopy images illustrating the subcellular localization of Nectin-4 in the OAW28 cell line. Cells were stained with (E) anti-Nectin-4 antibody (green) or (F) secondary antibody only as negative controls, F-actin of the cytoskeleton (gray), and DAPI (blue). Arrows highlight

cells exhibiting membrane-specific positive staining of Nectin-4. Scale bar, 10 μm. Abbreviations: T, tumor; N, normal tissue; HGSOC, high-grade serous ovarian carcinoma; LGSOC, low-grade serous ovarian carcinoma; EnOC, endometrioid ovarian carcinoma; CCOC, clear cell ovarian carcinoma; MOC, mucinous ovarian carcinoma; TPM, transcript per million; MFI, mean fluorescence intensity; DAPI, 4',6-Diamidino-2-phenylindole.

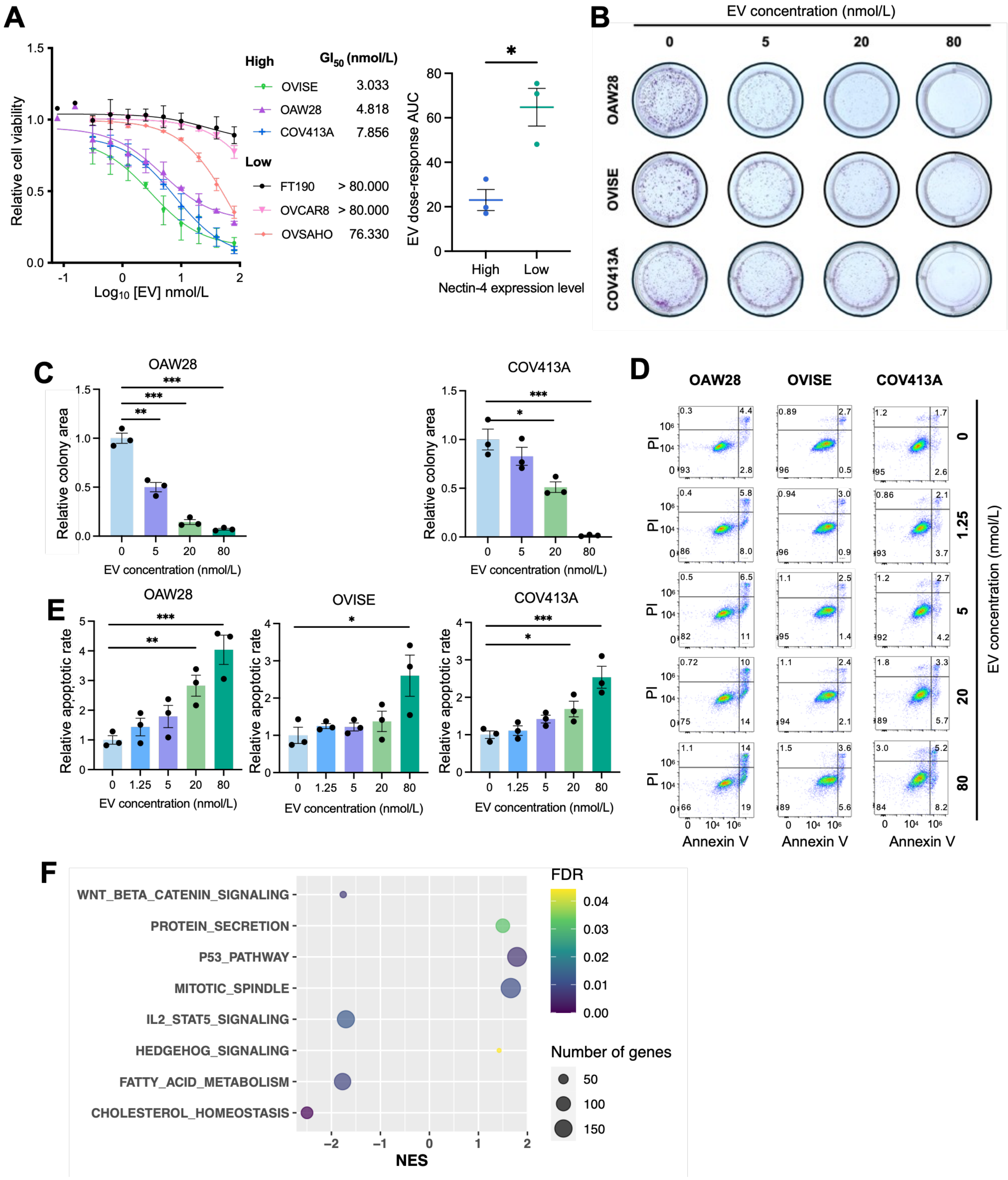

Fig. S2. ***In vitro*** **response to Nectin-4-directed antibody-drug conjugate in EOC cell lines. (A)** Left, dose-response curve of 7 EOC-relevant cell lines, including OVISE, OAW28, COV413A, FT190, OVCAR8 and OVSAHO, after treatment with 0-80 nmol/L EV for 7 days. Right, scatter plot depicting correlation between Nectin-4 expression level and EV sensitivity in the same set of EOC cell lines. AUC values (y-axis) are quantified based on Fig. S2A. Data are presented as mean ± SEM from three independent experiments. Based on their Nectin-4 expression (Fig. S1D), cell lines are classified as Nectin-4 high (OVISE, OAW28 and COV413A) or Nectin-4 low cell lines (FT190, OVCAR8 and OVSAHO), with corresponding GI_{50} values shown alongside each cell line. **(B)** Representative colony images of OAW28, OVISE and COV413A stained with crystal violet following 7 days of treatment with 0-80 nmol/L EV. **(C)** Quantification of colony formation in EOC cell lines (OAW28, and COV413A) in response to 0-80 nmol/L EV for 7 days. The colony area is quantified using ImageJ. Data are expressed as fold-change relative to control and presented as mean ± SEM of technical replicates (n = 3). **(D)** Representative flow cytometry plot of OAW28, OVISE and COV413A

stained with Annexin V-FITC (x-axis) and Propidium Iodide (PI) (y-axis) after treatment with 0-80 nmol/L EV. Numbers inside the plot indicate the percentage (%) of total cell population. **(E)** Cell apoptosis measured by Annexin V-FITC/PI flow cytometry in three EOC cell lines (OAW28, OVISE, and COV413A; n = 3) after 3 days of treatment with 0-80 nmol/L EV. Data are presented as fold-change relative to control and presented as mean ± SEM of three independent experiments. Statistical significance is determined by one-way ANOVA. **(F)** Summary of differential HALLMARK pathway enrichment in OVISE treated with 40 nmol/L EV for 3 days compared with non-treated control cells. X-axis, normalized enrichment score (NES). Statistical significance (FDR) of enrichment is indicated in colors. Statistical significance is determined by unpaired two-tailed Student's t-test (* $P < 0.05$, ** $P < 0.01$, *** $P < 0.001$). Abbreviations: EOC, epithelial ovarian cancer; AUC, area under the curve; EV, enfortumab vedotin; FDR, false discovery rate.

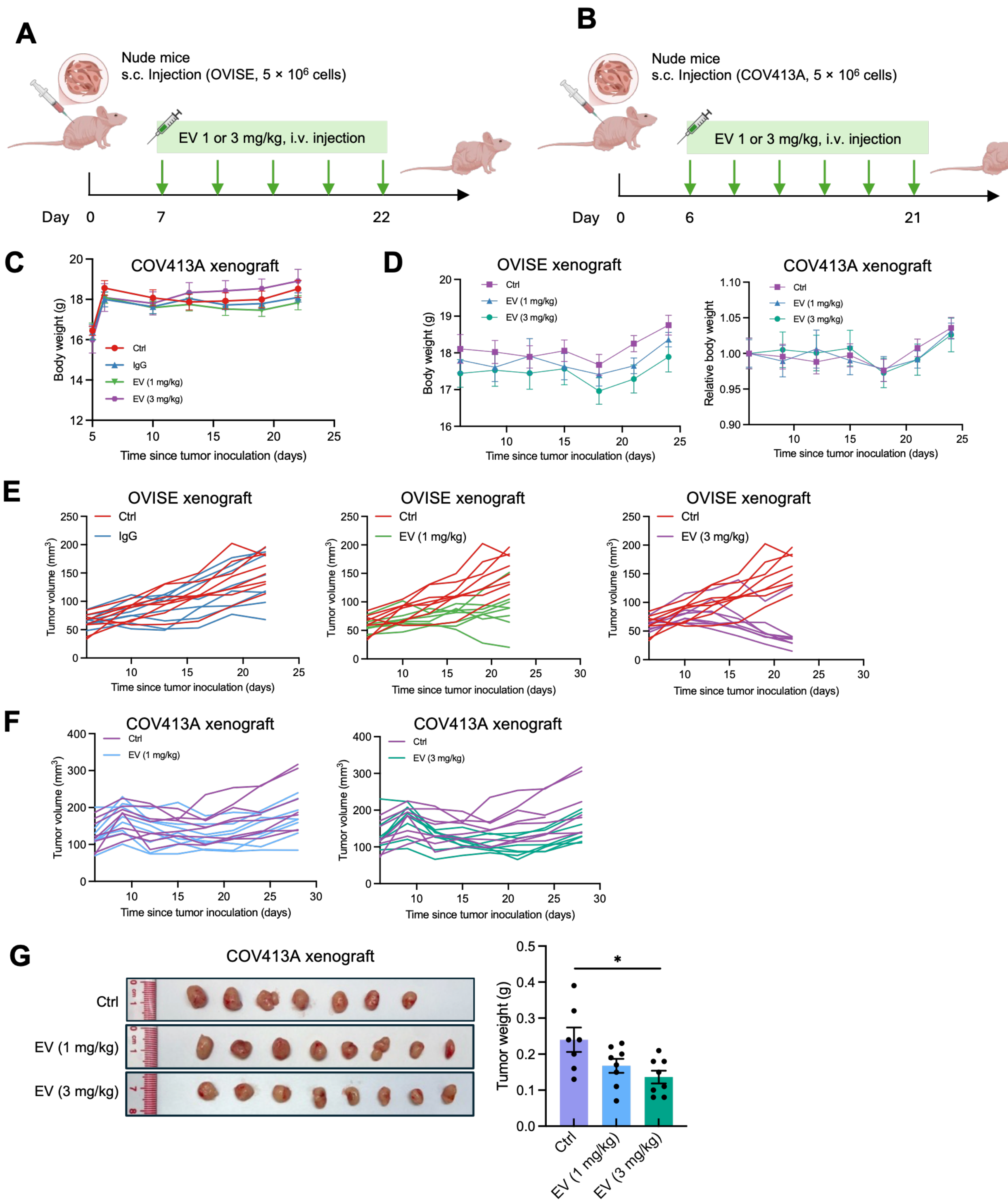

Fig. S3. *In vivo* efficacy of Nectin-4-directed ADC in EOC xenograft models. (A) Work scheme of EV monotherapy with OVISE xenograft model (n = 8 per group). Tumors stabilized on day 7 after inoculation and were chosen as the baseline. A total of 5 courses of saline, IgG or EV were administered intravenously, with a dose of 1 mg/kg or 3 mg/kg given every 3 days. Saline was used in the control group. **(B)** Work scheme of EV monotherapy with the COV413A xenograft model (n = 8 per group). Tumors stabilized on day 6 after inoculation and were chosen as the baseline. A total of 6 courses of EV were administered intravenously, with a dose of 1 mg/kg or 3 mg/kg given every 3 days. Saline was used in control groups. **(C)** Body weight of mice bearing COV413A-derived tumors throughout the treatment period. **(D)** Body weight (left) and relative body weight (normalized to day 6 after tumor inoculation; right) of mice bearing OVISE-derived tumors throughout the treatment period. **(E)** OVISE-derived tumor growth curves depicting tumor volume of individuals from the control groups (Ctrl or human IgG) and the treatment group (EV 1 mg/kg or 3 mg/kg). **(F)** COV413A-derived tumor growth curves depicting tumor volume of individuals from the control group and the 1 mg/kg EV (left) and 3 mg/kg EV (right) treatment group. **(G)** Left, tumor images from mice bearing COV413A-derived tumors after euthanasia at the study endpoint (day 35 post-inoculation). One mouse in the control group was euthanized

ahead of the study endpoint for tumor ulceration after receiving 5 courses of treatment, resulting in a final $n = 7$ for the control group and $n = 8$ for the treatment groups. Right, box plot showing final COV413A-derived tumor weight distribution among groups. Statistical significance is given by the unpaired two-tailed Student's t-test (* $P < 0.05$, ** $P < 0.01$). Abbreviations: ADC, antibody-drug conjugate; EOC, epithelial ovarian cancer; Ctrl, control; EV, enfortumab vedotin; s.c., subcutaneous.

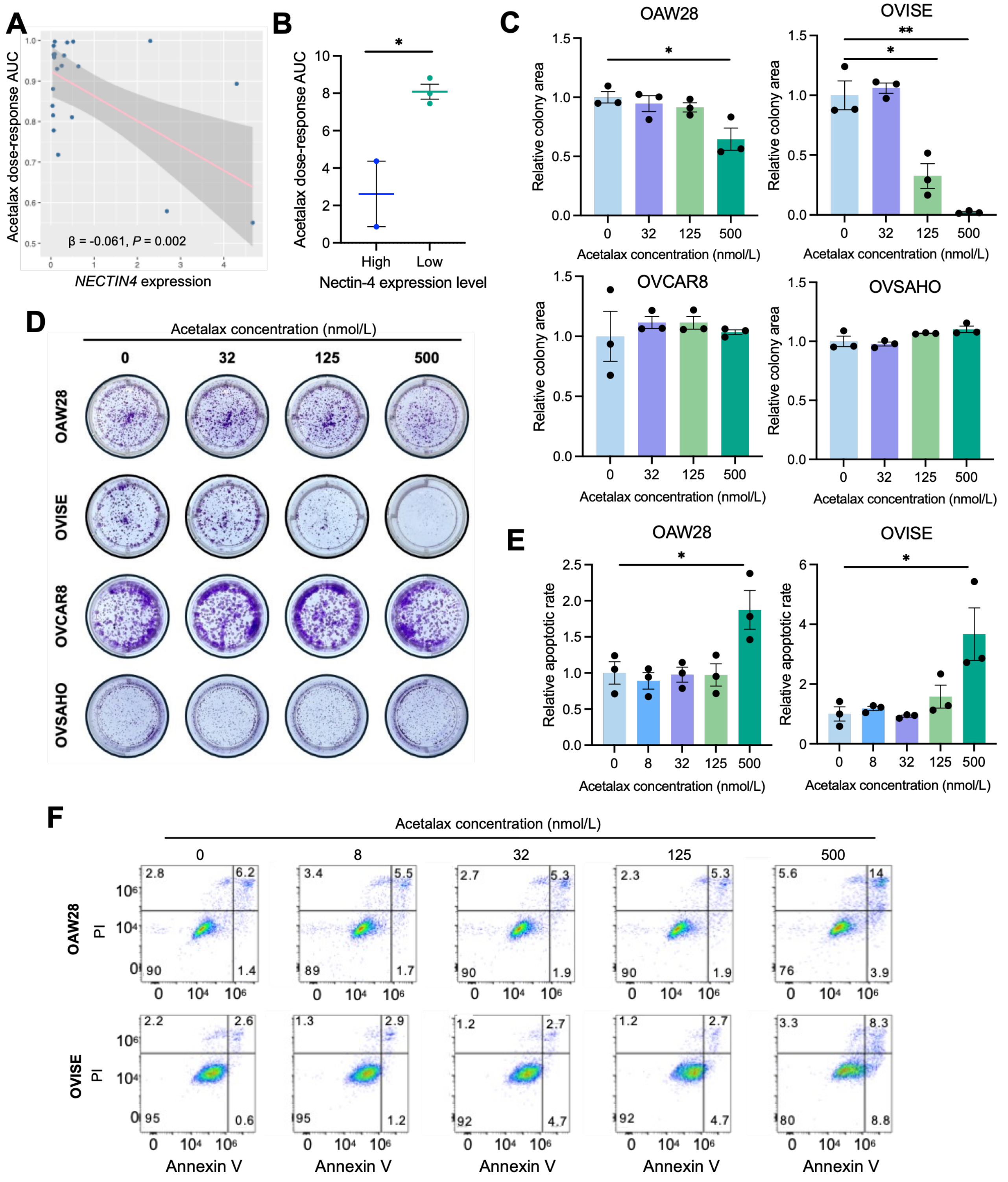

Fig. S4. ***In vitro*** **efficacy of acetalax in EOC cell lines. (A)** Scatter plot showing the correlation between *NECTIN4* mRNA expression in 22 EOC cell lines and their respective response to acetalax measured in AUC. Statistical significance is calculated using linear regression. **(B)** Scatter plot depicting correlation between Nectin-4 expression level and acetalax sensitivity in 5 EOC cell lines including OVISE, OAW28, FT190, OVCAR8 and OVSAHO. GI_{50} values (y-axis) are quantified based on Fig. 1I. Cell lines are categorized into high (OVISE, OAW28) and low (FT190, OVCAR8 and OVSAHO) groups based on Nectin-4 expression levels (Fig. S1D). **(C)** Quantification of colony formation in OAW28, OVISE, OVSAHO and OVCAR8 in response to 0-500 nmol/L acetalax for 7 days. Data are expressed as fold-change relative to control and presented as mean ± SEM from three independent experiments. **(D)** Representative colony images of OAW28, OVISE, OVSAHO and OVCAR8 cell lines stained with crystal violet following 7 days of treatment with 0-500 nmol/L acetalax. **(E)** Cell apoptosis measured by Annexin V-FITC/PI flow cytometry in OAW28 and OVISE after 3 days of treatment with 0-500 nmol/L acetalax. Data are presented as fold-change relative to control and presented as mean ± SEM of three independent experiments. **(F)** Representative flow

cytometry plot of OAW28 and OVISE stained with Annexin V-FITC (x-axis) and PI (y-axis) after treatment with 0-500 nmol/L acetalax for 3 days. Numbers inside the plot indicate the percentage (%) of total cell population. The statistical significance is determined by an unpaired two-tailed Student's t-test (* $P < 0.05$, ** $P < 0.01$, *** $P < 0.001$). Abbreviations: PI, propropidium iodide; FITC, fluorescein isothiocyanate.

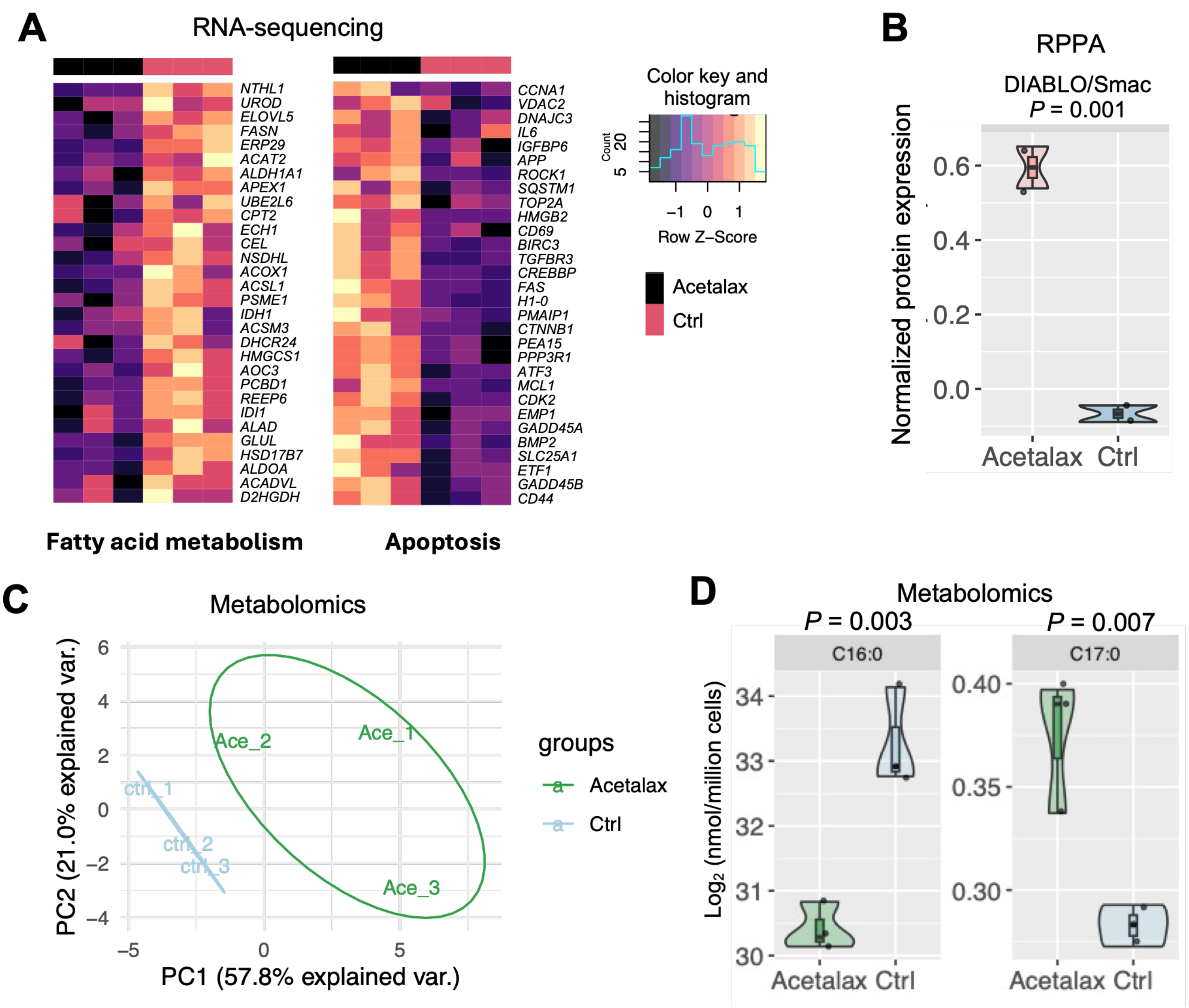

Fig. S5. Biological pathways dysregulated by acetalax in OVISE cell line. (A) Heatmap illustrating gene expression patterns involved in fatty acid metabolism (left) and the apoptosis (right) pathway in OVISE cells treated with 100 nmol/L acetalax for three days. **(B)** Violin plot showing altered expression of diablo IAP-binding mitochondrial protein (DIABLO) in OVISE cells treated with 100 nmol/L acetalax for three days. Y-axis, normalized protein expression. **(C)** Principal component analysis plot showing the distribution of global fatty acids in 100 nmol/L acetalax-treated and non-treated OVISE cells (Ctrl) for three days. **(D)** Violin plot illustrating the dysregulation of fatty acids in 100 nmol/L acetalax-treated OVISE for three days by targeted metabolomics profiling. Y-axis, nmol/million cells in $\log_2$ ratio. Abbreviations: Ctrl, control; PC, principal component; C16:0, palmitic acid; C17:0, Heptadecanoic acid.

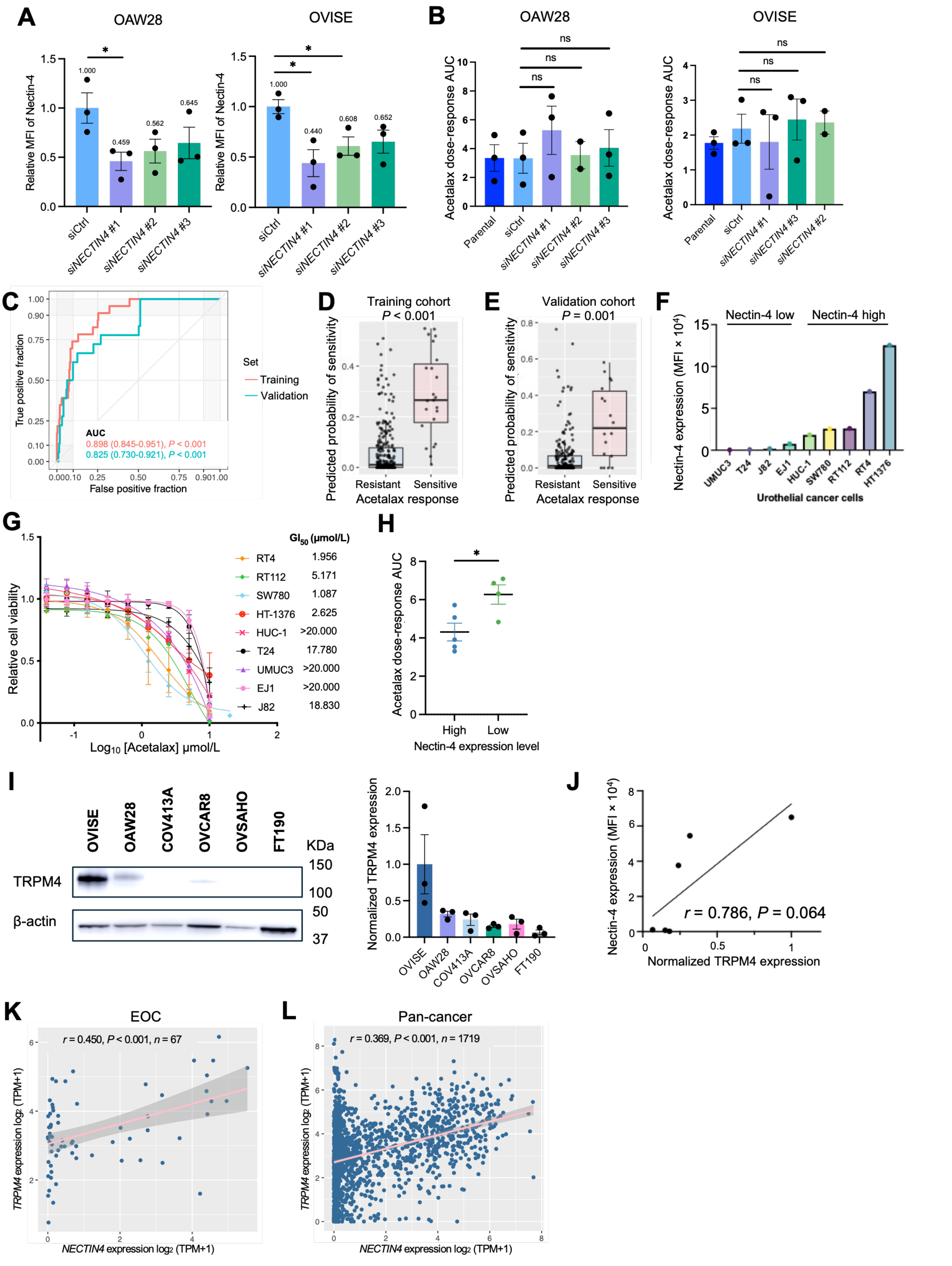

Fig. S6. Nectin-4 and acetalax sensitivity in cancer cells. (A) Bar chart showing Nectin-4 knockdown (with three independent siRNAs) efficiency in OAW28 and OVISE. Nectin-4 expression in cell lines was measured by flow cytometry and presented as the relative mean fluorescence intensity (MFI) compared to the non-targeting control siRNA (siCtrl) group. **(B)** Bar chart showing dose-response AUC of OAW28 and OVISE cells transfected with either control siRNA or siRNA targeting *NECTIN4*, followed by treatment with 0, 0.039, 0.078, 0.157, 0.313, 0.625, 1.250, 2.500,

5.000 or 10.000 μmol/L acetalax for 3 days. Dose-response area under the curve (AUC) values were estimated from cell viability assays. **(C)** ROC curve demonstrating the ability of *NECTIN4* transcript levels to predict acetalax sensitivity in 540 cancer cell lines from the DepMap database. The 540 cell lines were randomly divided into training (n = 270; red line) and validation (n = 270; blue line) sets. A generalized linear model incorporating *NECTIN4* expression and cell lineage was fit to dichotomized acetalax sensitivity in the training set, and the resulting model was used to predict acetalax response in the validation set. AUC and 95% CI generated by DeLong method are indicated. **(D-E)** Predicted probability of acetalax sensitivity (y-axis), derived from the logistic regression model in Fig. S6C, plotted against observed acetalax response (x-axis). The model was fitted with *NECTIN4* expression as the predictor, and acetalax sensitivity as a binary outcome. Predicted values are probabilities obtained on the response scale, where higher values indicate a greater predicted likelihood of sensitivity. Cell lines from the DepMap database (n = 520) were randomly assigned in equal proportion to a training set (D, n = 270) and a held-out validation set (E, n = 270). Boxes show median and interquartile range; whiskers extend to 1.5× interquartile range; individual cell lines are overlaid as points. **(F)** Membrane Nectin-4 expression in 9 urothelial cancer cell lines (UMUC3, T24, J82, EJ1, HUC-1, SW780, RT112, RT4, and HT1376) measured by flow cytometry. **(G)** Cell viability response of urothelial cancer cell lines to acetalax. The corresponding GI_{50} value are indicated alongside each cell line. **(H)** Acetalax sensitivity in 9 urothelial cancer cell lines stratified by Nectin-4 expression. Bar plot showing the AUC for urothelial cancer cell lines categorized as Nectin-4-high and Nectin-4-low groups based on Fig. S6F. **(I)** Representative Western blotting image (left) and quantifications (right) showing TRPM4 expression in a panel of EOC cell lines (n = 3). β-actin was used as the internal control. **(J)** Scatter plot illustrating the correlation between TRPM4 protein expression (x-axis) and Nectin-4 protein expression (y-axis) in six EOC cell lines from Figs. S6I and S1D. **(K-L)** Scatter plot showing correlation between *NECTIN4* and *TRPM4* mRNA expression in EOC cells (K) and pan-cancer cells (L) from the DepMap database. Correlation coefficient and P-value is given by Pearson method in Figs. S6K-L. Data are presented as mean ± SEM from three independent experiments. Statistical significance is determined by an unpaired two-tailed Student's t-test (ns (non-significant) P > 0.05, * P < 0.05, ** P < 0.01, *** P < 0.001). Abbreviations: AUC, area under curve; CI, confidence interval; MFI, mean fluorescence intensity.

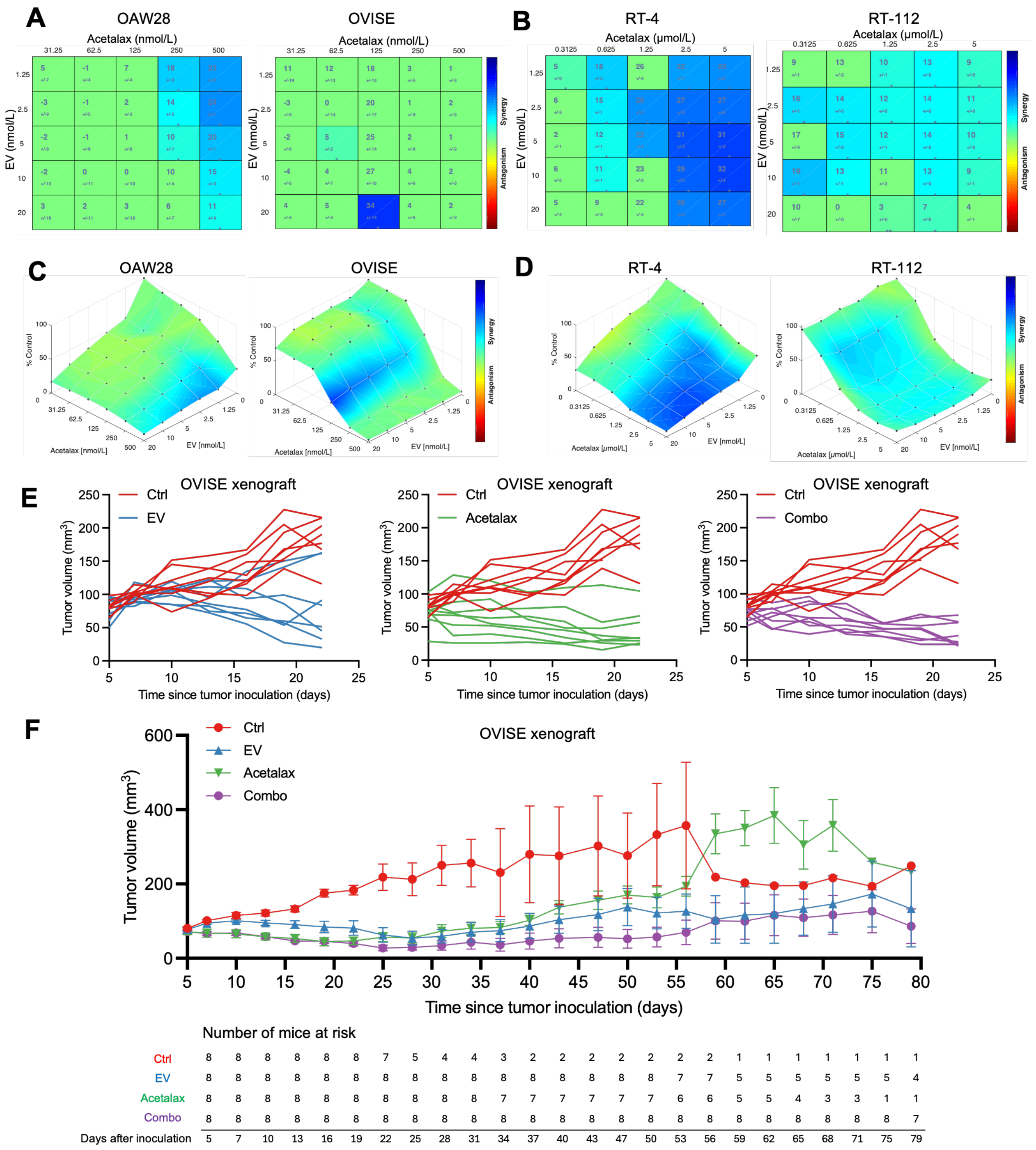

Fig. S7. Therapeutic efficacy of EV and acetalax combination in Nectin-4-high EOC. (A-B) Heatmaps depicting synergy scores in (A) OAW28, OVISE, (B) RT-4 and RT-112 ($n = 3$, representative data shown) treated with 0-20 nmol/L EV and 0-5 μmol/L acetalax for 7 days. Drug synergistic effects were analyzed by Combenefit software using the HSA model. Heatmaps represent the combined effects of the two drugs. Values within the heatmap indicate the synergy score: blue (>0) indicates synergy, green (=0) indicates additivity, and red (<0) indicates antagonism. **(C-D)** Dose-response matrices depicting cell viability mapped with synergy scores in (C) OAW28, OVISE, (D) RT-4 and RT-112 ($n = 3$, representative data shown) treated with 0-20 nmol/L EV and 0-5 μmol/L acetalax for 7 days. Relative cell viability, including the single-agent response (0 concentration control for each drug), is indicated on the y-axis. Drug synergistic effects were analyzed by Combenefit software using the HSA model. Heat maps represent the combined effects of the two drugs. Values within the heatmap indicate the synergy score: blue (>0) indicates synergy, green (=0) indicates additivity, and red (<0) indicates antagonism. **(E)** OVISE-derived tumor growth curves depicting tumor volume of individuals in the saline control, 1-3 mg/kg escalated EV, 50 mg/kg acetalax and combination group, as shown in Fig.

1M (*n* = 8). **(F)** Top, OVISE-derived tumor volume measured every three days from baseline until day 79 post-inoculation. Bottom, the number of mice at risk for tumor measurement at each time point. Abbreviations: EV, enfortumab vedotin; HSA, highest single agent; combo, combination.

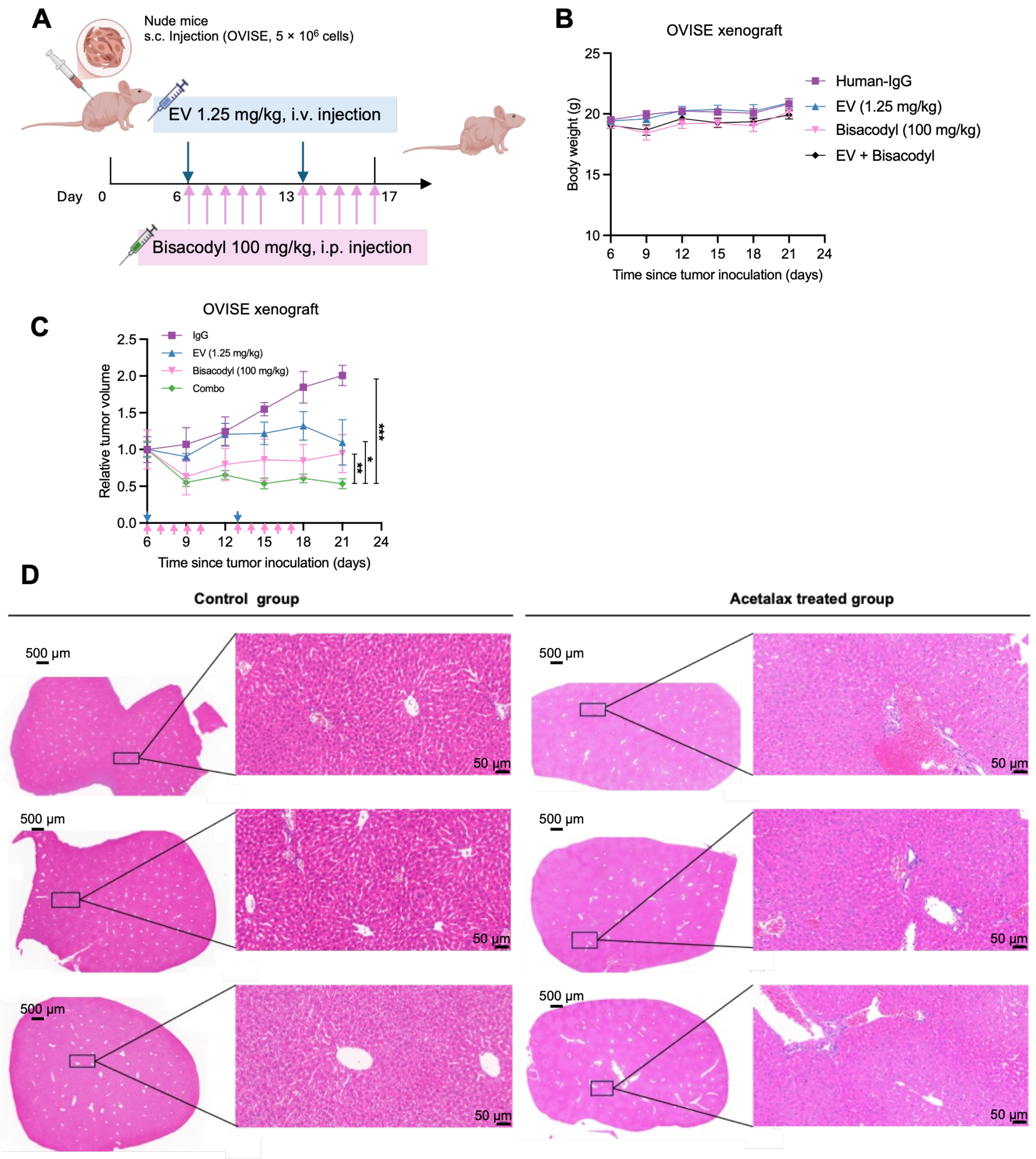

Fig. S8. ***In vivo*** **efficacy of EV, bisacodyl and their combination in OVISE-derived xenograft model.**

(A) Work scheme of the *in vivo* experiment with OVISE model. Tumors stabilized on day 6 after inoculation and were chosen as the baseline. In single-agent group, a total of 2 courses of 1.25 mg/kg EV were given intravenously once per week. A total of 10 courses of bisacodyl 100 mg/kg were given for 5 days followed by 2 days break. Identical dosing schedules were applied to combination arms. Human IgG (1.25 mg/kg) was used in control groups. **(B)** Body weight of mice bearing OVISE-derived tumors within 21 days after tumor inoculation. **(C)** OVISE-derived tumor volume measured every three days from baseline until day 21 post-inoculation. EV (1.25 mg/kg, once per week) and bisacodyl (100 mg/kg, once per day for 5 days followed by 2 days break) were administered as single agents or in combination according to the schedule outlined in Fig. S8A. Arrows indicate the treatment schedule for EV at 1.25 mg/kg (top) or bisacodyl at 100 mg/kg (bottom). **(D)** Representative hematoxylin and eosin staining images of livers from mice bearing OVISE-derived tumors treated with saline control (left panel) or daily 50 mg/kg acetalax treatment for 15 days (right

panel). *n* = 3 per group. Statistical significance is determined by an unpaired two-tailed Student's t-test (* $P < 0.05$, ** $P < 0.01$, *** $P < 0.001$). Abbreviations: EV, enfortumab vedotin; i.p., intra-peritoneal; i.v., intravenous.

Reference

1. Perets R, Wyant GA, Muto KW, Bijron JG, Poole BB, Chin KT, et al. Transformation of the Fallopian Tube Secretory Epithelium Leads to High-grade Serous Ovarian Cancer in Brca;Tp53;Pten Models. Cancer cell. 2013;24(6):751-.
2. Di Veroli GY, Fornari C, Wang D, Mollard S, Bramhall JL, Richards FM, et al. Combenefit: an interactive platform for the analysis and visualization of drug combinations. Bioinformatics. 2016;32(18):2866-8.
3. Shehwana H, Kumar SV, Melott JM, Rohrdanz MA, Wakefield C, Ju Z, et al. RPPA SPACE: an R package for normalization and quantitation of Reverse-Phase Protein Array data. Bioinformatics. 2022;38(22):5131-3.
4. Reich M, Hannig C, Al-Ahmad A, Bolek R, Kümmerer K. A comprehensive method for determination of fatty acids in the initial oral biofilm (pellicle). Journal of Lipid Research. 2012;53(10):2226-30.
